# Effect of Cooling Blanket on the Heat Stress of Horses in Hot and Humid Environments

**DOI:** 10.3390/ani12192505

**Published:** 2022-09-20

**Authors:** Yuki Ojima, Suzuka Torii, Yosuke Maeda, Akihiro Matsuura

**Affiliations:** Department of Animal Science, School of Veterinary Medicine, Kitasato University, 23-35-1, Higashi, Towada, Aomori 034-8628, Japan

**Keywords:** heat stress, horse, cooling, welfare, ice vest, cortisol, skin surface temperature, stall

## Abstract

**Simple Summary:**

Heat stress is serious problem for livestock. While riding horses and racehorses spend the majority of their days in stalls, there are few reports on effective methods for reducing stall heat stress. The aim of the present study was to evaluate the effectiveness of an ice horse blanket in hot and humid environments. Twenty healthy horses were measured first without the blanket (C) and then measured with the blanket (IB), or vice versa, in a cross-over trial. The blanket was designed to keep cooling the front back, the rear back, and the loin. The skin surface temperature of the front back was decreased with cooling time in IB, whereas it was not changed in C. Similarly, respiratory rate and plasma cortisol level also decreased only in IB. The blanket used in the present study had the advantage of allowing for gentle cooling of the horse’s body without the use of water or fans. Applying this methodology should enable effective reduction of heat stress not only in horses but also in other mammals kept in barns.

**Abstract:**

Heat stress is serious problem for livestock. While riding horses and racehorses spend the majority of their days in stalls, there are few reports on effective methods for reducing stall heat stress. The aim of the present study was to evaluate the effectiveness of an ice horse blanket in hot and humid environments. Twenty healthy horses were measured first without the blanket (C) and then measured with the blanket (IB), or vice versa, in a cross-over trial. The blanket was designed to keep cooling the front back, the rear back, and the loin. Skin surface temperature, respiratory rate, rectal temperature, and plasma cortisol level in both C and IB were measured at 10:00, 12:30, and 15:00. The skin surface temperature of the front back was decreased with cooling time in IB, whereas it was not changed in C. Similarly, heart rate, respiratory rate, and plasma cortisol level also decreased only in IB. The blanket used in the present study had the advantage of allowing for gentle cooling of the horse’s body without the use of water or fans. Applying this methodology should enable effective reduction of heat stress not only in horses but also in other mammals kept in barns.

## 1. Introduction

Global warming has been a point of contention for scientists, politicians, and environmentalists. Global warming is a gradual process that threatens to have serious consequences such as rising sea levels, crop failure and famine, changes in global rainfall patterns, changes in plant and animal populations, and serious health consequences [1]. According to Hu et al. [2], the global mean surface temperature (GMST) has increased rapidly in recent decades, and global warming has become a well-known topic. While global warming appears to have slowed in the decade from 2000 to 2014, the GMST reached an historic high in 2015. Global warming continued in 2021, and temperatures around the world and Japan remained consistent with the Japan Environmental Ministry’s guidelines [3]. As global warming continues, temperatures will continue to rise, increasing the risk of developing heat-related diseases such as heat stroke.

Indeed, heat is a major health issue for livestock. Park et al. [4] stated that heat stress in industrial animals decreased productivity and immunity. According to Chaiybutr et al. [5], heat stress reduces milk production in cattle. Heat stress due to either climate or exercise, or both, has been shown to increase the risk of heat stroke in horses [6]. Horses have a high metabolic rate but a small surface area for heat dissipation, as described by Janczarek et al. [7]. As a result, horses must be actively cooled, even more so in hot environments. Studies of heat in horses often focus on the effects of exercise and in the paddock [6,7,8,9]. However, there are few reports on the stall where horses spend the majority of their lives.

Fans, mist fans, evaporative cooling, and sprinklers are well-established ways to cool cattle in heat [5,10,11,12,13]. Humans frequently use fans and air conditioners as heat protection devices [14,15]. In the case of horses, mist curtains, fans, and cold ice water poured over the horse’s body are used to reduce heat stress [7,8,16]. However, it is not appropriate to use water in the stall, as it is widely believed that water causes horses to slip, they injure themselves, and if a horse spends a long time in the stall with its hoof wet, bacteria and fungus will develop on the hoof, making it unsanitary and in fact, the insanitary condition of it can cause hoof disease such as white line disease and thrush [17,18].

Takahashi et al. [8] reported that cooling with two fans was less effective than cooling with water in Thoroughbreds following exercise. McGill et al. [19], who evaluated the treatment by drenching the horses’ bodies with a fan following exercise, found no effect on heart rate, respiratory rate, and rectal temperature. According to these findings, fans alone would be insufficient to cool the horses.

Recently, a new method of attaching ice packs to clothing has been developed for humans. Zare et al. [20] indicated that the ice vest had a positive effect on perceptual strain and surface temperature in humans. Furthermore, Luomala et al. [21] showed that the ice vest decreased a part of skin surface temperature. Hasegawa et al. [22] proved that the ice vest significantly decreased mean skin temperature and heart rate in humans. Horses have the highest sweating rates of any endotherm, approximately double the maximum sweating rates of humans [23]. Additionally, Collier et al. [23], reported that cutaneous and respiratory evaporative heat loss contributed approximately 71% and 29%, respectively, to total evaporative heat loss on horses in hot and humid environments. On the other hand, Bilgili et al. [24], reported that cutaneous and respiratory evaporative heat loss contributed approximately 90% and 10%, respectively, to total evaporative heat loss in humans. These two studies, as well as others [7,8], demonstrate that horses and humans have comparable thermal regulation. Due to the similarities in thermal regulation between horses and humans, the ice vest could be used to provide heat protection for horses. Because the ice vest technique does not require water or wind, it should be effective for horses in stalls.

There is a growing interest in animal welfare at the moment. Animal welfare includes the five freedoms of animals comprising: freedom from hunger, malnutrition, and thirst; freedom from fear and distress; freedom from heat stress or physical discomfort; freedom from pain, injury, and disease; and freedom to express normal patterns of behaviour [25]. Improving the thermal environment of horses is deemed critical because it results in increased freedom from heat stress or physical discomfort, as well as freedom from pain, injury, and disease. Indeed, if the heat environment is not improved, the risk of heat stroke and associated complications increases in horses [7,8].

The aim of the present study was to determine whether an ice blanket was effective on heart rate (HR), respiratory rate (RR), skin temperature (SK), rectal temperature (RT), and plasma cortisol in horses, particularly in the stall, in hot and humid environments.

## 2. Materials and Methods

The present study was conducted at Kitasato University’s veterinary school’s stable and a private horseback riding club. Both facilities were located in Towada city (Aomori prefecture, Japan), at latitude 40.4° N and longitude 141.1° E, respectively. The Kitasato University School of Veterinary Medicine’s Ethics Committee approved this study (Ref No. 21-041).

### 2.1. Horses

Seven Thoroughbreds (6 geldings and 1 female) and 13 cross-breeds were used (8 geldings and 5 female). Clinically sound, the horses were routinely used as riding horses. The age (mean ± SD) was 11.8 ± 5.8. On the experimental days, the horses were not exercised and were resting in their stalls.

### 2.2. Ice Horse Blankets

**Figure 1:**
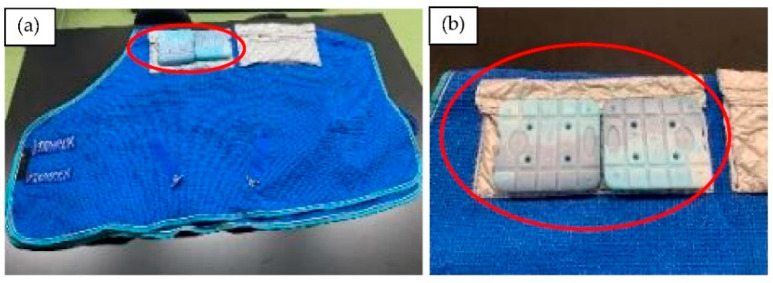
The ice horse blanket consisted of a thin horse blanket, four bags, and 8 ice packs. (**a**) One side of an ice horse blanket is shown. (**b**) Each cold-sheet bag could contain two ice packs.

The ice horse blanket consisted of a thin horse blanket made of rag waffle fabric (WAFFLE RUG, Oriental-Sophie CO., Tochigi, Japan), four cold-sheet bags (Cooling and Warming Sheets, Nemuritti LLC., Tochigi, Japan) and 8 ice packs (melting point 0 °C, 750 g, 170 × 195 × 30 mm per ice pack, CAPTAIN STAG, NATURAMU Co., Osaka, Japan, Figure 1a,b). Each cold-sheet bag is capable of holding two ice packs (Figure 1b). The cold sheets have properties which do not conduct heat easily (heat retaining property), the cold-sheet bags which are made from the cold sheets also have the same property. The ice horse blanket weighs approximately 9 kg in total.

It was designed to keep cooling the 3 sites of the horse’s body: the front back, the rear back, and the loin. To ensure that they would not overcool the horse’s body, a preliminary study determined that the contact area would maintain a temperature of approximately 20 °C. In addition, preliminary experiments proved that the temperature of the contact area did not change within at least five hours under the sun. According to the manufacturer’s instructions, the ice pack should be kept below freezing for at least 2 h in an environment of 30 °C.

### 2.3. Experimental Design

**Figure 2:**
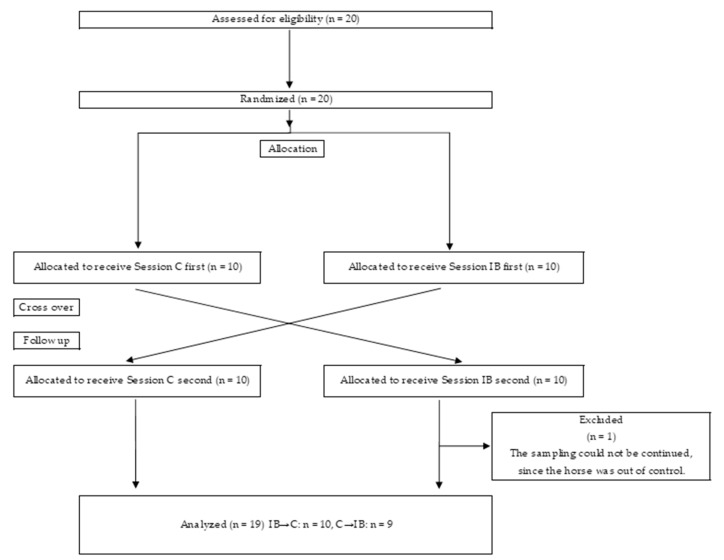
Flow diagram of the cross-over trial.

To eliminate the effect of order on treatment, a cross-over trial was conducted, as shown in Figure 2. In one group, horses were first measured without a blanket (C) and then treated with an ice horse blanket (IB). In another group, horses were first measured with an ice blanket (IB) and then without the blanket (C). We did not put any blanket on the horses in the C measurements, as the preliminary study predicted that the horses would sweat excessively and exhaustion would occur in the blanket without ice packs. Additionally, to eliminate the effects of continuous days of experimentation, a gap of at least one day between measurement dates was allowed.

### 2.4. Procedure

**Figure 3:**
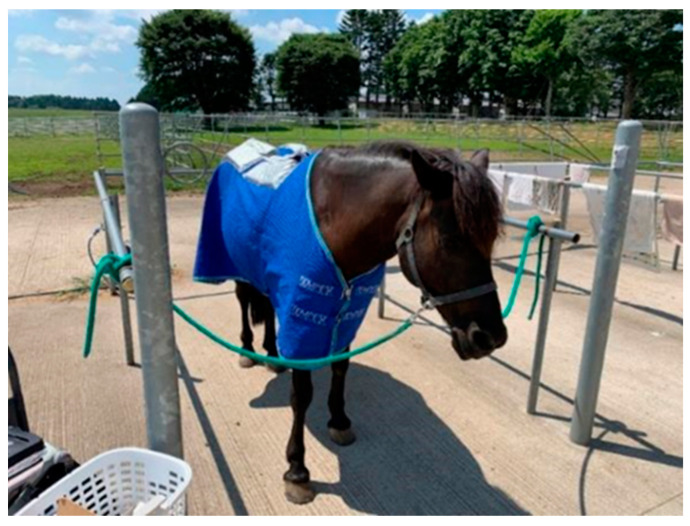
In the IB groups, the horses were equipped with ice horse blankets.

Five variables were measured sequentially: (1) skin surface temperature; (2) heart rate, HR; (3) respiratory rate, RR; (4) rectal temperature, RT; and (5) plasma cortisol. The order of the measurements was chosen to minimize the effects of the stimuli. Sampling was always carried out while a horse was clipped to a wash rack without a blanket. After the first sampling (10:00), the ice horse blanket was placed on IB’s horse (Figure 3). The horse was then returned to its stall. The horse was sampled three times per treatment. The sampling times were 10:00, 12:30, and 15:00. Sampling was always carried out without the use of a blanket. Because the cooling effect of the ice packs might decrease faster than expected due to the body temperature of the horses, all eight ice packs were replaced at 12:30 with freshly frozen ones as a precaution. One sampling per horse took less than 15 min.

### 2.5. Physiological Measures

**Figure 4:**
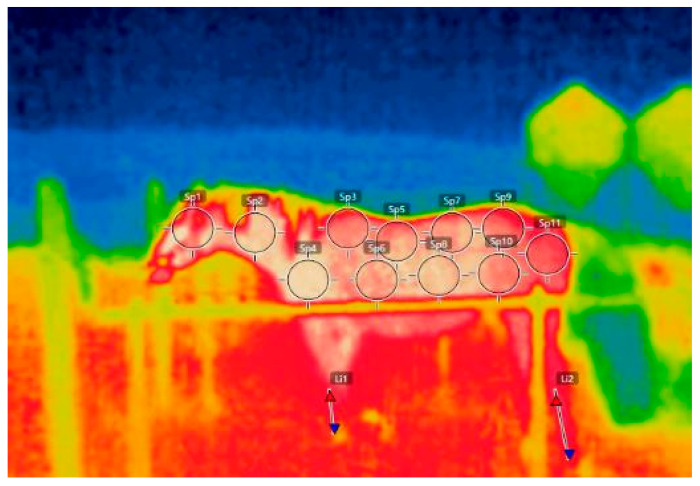
The 13 body sites; (SP1; head, SP2; neck, SP3; withers, SP4; shoulder, SP5; front back, SP6; front abdomen, SP7; rear back, SP8; rear abdomen, SP9; loin, SP10; thigh, SP11; buttock, li1; fore-cannon, Li2; rear-cannon).

Skin surface temperature, HR, RR, and RT were measured at 10:00, 12:30, and 15:00. A lead rope was clipped to the horse’s halter for the measurements, and the horse was positioned to stand in areas where horses are constantly cared for with brushes and other items. A thermal camera was used to determine the temperature of the horses’ skin surface on the left (FLIR CX-Series Compact Thermal Imaging Camera, FLIR Co., Wilsonville, OR, USA). Skin surface temperature was always photographed, immediately after the ice horse blanket was removed. The skin surface temperature at 13 body sites was measured using a slight modification of the method described by Wilk et al. [26] and Westermann et al. [27] (Figure 4). Skin surface temperatures had to be obtained including the horse’s entire body at one time immediately after its blanket was removed because, if a time difference occurred, the temperature could change and would not be accurate. The distance required to capture the horse’s entire body at one time was 2 m away from the horse. Obtaining 2 m was difficult in the stalls or the stables. Therefore, horses were clipped to wash rucks outside the stables and skin surface temperatures were obtained at the place. At once, Sampling of other Variables was also carried out continuously at the same place. The HR was determined by using a stethoscope (Classic ΙΙ S. E. Stethoscope, 3M Japan Co., Tokyo, Japan). The RR was determined visually by observing flank movement. RT was obtained by using a digital thermometer (CITIZEN CT 422, CITIZEN Inc., Tokyo, Japan) inserted 6.5 cm into the rectum.

### 2.6. Cortisol Assays

At 10:00, 12:30, and 15:00, blood samples (5 mL each) were collected via jugular venipuncture into an evacuated vial containing heparin (VP-H050k, TERUMO Inc., Tokyo, Japan). They were placed on ice and centrifuged (2000× *g*, 10 min) to separate the plasma. Within 2 h, the plasma samples were stored at −30 °C until analyses. Cortisol levels in the plasma were determined in duplicate using the Cortisol Enzyme Immunoassay Kit (K003-H5, Arbor Assays Co., Ann Arbor, MI, USA) according to the manufacturer’s protocol.

### 2.7. Statistical Analysis

Data from one gelding horse were excluded as the horse was out of control and the sampling could not be continued (Figure 2). Seven Thoroughbreds and twelve crossbreds were used in the statistical analysis. The age (mean ± SD) was 12.2 ± 5.7. Two-way repeated measures analysis of variance (ANOVA) was used to analyze the interaction between the treatment and time (treatment × time), the main effect of treatment (C vs. IB) and the main effect of time (10:00 vs. 12:30 vs. 15:00). When a significant main effect in time or a significant interaction was found, one-way repeated ANOVA was used to determine the difference between the three time points (simple main effect in time). When a significant interaction was found, one-way repeated measures ANOVA was also used to determine the simple main effect in treatment. If the ANOVA was significant, Tukey’s multiple comparison test was used to identify the difference between different time points. The amount of change in concentration between the time of measurement and 10:00 (Δ Plasma cortisol) was also calculated for plasma cortisol only. Statistical analysis was performed almost identically also for Δ plasma cortisol. However, the multiple comparisons were not performed for the time of Δ plasma cortisol, as the number of levels on main factor in time of Δ plasma cortisol was only two (12:30–10:00, 15:00–10:00).

Correlation between surface temperatures of the 3 sites of horse’s body (front back, rear back, and loin) and 3 measurement variables (HR, RR, and plasma cortisol) was analyzed with a Pearson’s product moment correlation coefficient. The correlation analysis was carried out for both, C and IB groups (both groups; n = 19 × 6, C group; n = 19 × 3 and IB group; n = 19 × 3). The differences between means correlations were considered significant at *p* < 0.05 and considered to be a tendency at *p* < 0.1.

### 2.8. Weather Condition

The present study was conducted on days when the weather forecast indicated that the ambient temperature would exceed 25 °C. The atmospheric conditions were estimated using the environmental meter (ENVIRONMENTAL METER AHLT-100, AS ONE Inc., Santa Clara, CA, USA. The ambient temperature (Tamb), humidity (RH), and the wind speed were based on the device set in front of a specific stable located in the center of the stable. To avoid differences in the management environment between normal and measurement days, on measurement day, the fan was left on for 9 horses that normally have a fan in their stall. The temperature–humidity index (THI) was calculated from Tamb (°C) and RH (%) in the present study, based on the formula used by Holcomb et al. [9] and Schütz et al. [28]:

THI = (1.8 × Tamb + 32) − [(0.55 − 0.0055 × RH) × (1.8 × Tamb − 26)].

Tamb, RH, and THI at each time point of C and IB are shown in Table 1 and Table 2. Values are expressed as mean ± SE. There was no significant interaction for Tamb between time and treatment. The main effect of treatment was not significant. The main effect of time was significant (*p* < 0.05) and Tukey’s test revealed that the Tamb at 12:30 was significantly higher than that at 10:00. Tamb at 15:00 tended to be higher than that at 10:00. There was no significant interaction for RH between time and treatment. The main effect of treatment was not significant. The main effect of time was significant (*p* < 0.05) and Tukey’s test revealed that the RH at 12:30 was significantly lower than that at 10:00. There was no significant interaction for THI between time and treatment. The main effect of treatment was not significant. The main effect of time was significant (*p* < 0.05) and Tukey’s test revealed that the THIs at 12:30 and 15:00 were significantly higher than that at 10:00.

There was no significant interaction for the wind speed between time and treatment. The main effects of time and treatment were not significant. The wind speed was 0 m/s, but the wind speed by the fan was 0.1–1.5 m/s.

## 3. Results

### 3.1. Physiological Measures

The skin surface temperature at each time point of C and IB are shown in Table 3, Table 4 and Table 5. There was significant interaction for the skin surface temperature on front back between treatment and time (*p* < 0.05). The simple main effects of treatment were significant at 12:30 and 15:00 (*p* < 0.05). In other words, the skin surface temperatures (front back) in the IB at 12:30 and 15:00 were significantly lower than those of C (*p* < 0.05). The simple main effect of time was significant only in the IB (*p* < 0.05). In the IB group, the skin surface temperature (front back) at 15:00 was significantly lower than that at 10:00 (*p* < 0.05). There was no significant interaction for the skin surface temperature on rear back between treatment and time. However, the *p*-value of the interaction tended to be significant (*p* = 0.058). The simple main effects of treatment were significant at 12:30 and 15:00 (*p* < 0.05). In other words, the skin surface temperatures (rear back) in the IB at 12:30 and 15:00 were significantly lower than those of C (*p* < 0.05). The simple main effect of time was not significant. However, the *p*-value of the main effect of time tended to be significant only in the IB group (*p* = 0.061). In the IB group, the skin surface temperature (rear back) at 15:00 tended to be lower than that at 10:00 (*p* = 0.056). There was significant interaction for the skin surface temperature on loin between treatment and time (*p* < 0.05). The simple main effects of treatment were significant at 12:30 and 15:00 (*p* < 0.05). In other words, the skin surface temperatures (loin) in the IB at 12:30 and 15:00 were significantly lower than those of C (*p* < 0.05). For the skin surface temperature on fore-cannon, the main effect of time was significant (*p* < 0.05) and Tukey’s test revealed that the skin surface temperature (fore-cannon) at 12:30 was significantly higher than that at 10:00 (*p* < 0.05). For the skin surface temperature on rear-cannon, the main effect of time was significant (*p* < 0.05) and Tukey’s test revealed that the skin surface temperature (rear-cannon) at 12:30 was significantly higher than that of 10:00 (*p* < 0.05). There was neither significant interaction for the skin surface temperature in other sites of the horse’s body between treatment and time, nor the main effects of treatment and time.

HR, RR, and RT at each time point of C and IB are shown in Table 6, Table 7 and Table 8. There was significant interaction for HR between treatment and time (*p* < 0.05). The simple main effects of treatment were significant at 12:30 and 15:00 (*p* < 0.05). In other words, HRs in the IB at 12:30 and 15:00 were significantly lower than those of C (*p* < 0.05). The simple main effect of time was significant only in the IB (*p* < 0.05). In the IB group, the HRs at 12:30 and 15:00 were significantly lower than that of 10:00 (*p* < 0.05). There was significant interaction for RR between treatment and time (*p* < 0.05). The simple main effects of treatment were significant at 12:30 and 15:00 (*p* < 0.05). In other words, RRs in the IB at 12:30 and 15:00 were significantly lower than that of C (*p* < 0.05). The simple main effect of time was significant only in the IB (*p* < 0.05). In the IB group, the RRs at 12:30 and 15:00 were significantly lower than that of 10:00 (*p* < 0.05). In RT, the main effect of treatment was significant, and RT was higher in the IB than C (*p* < 0.05). The main effect of time was not significant. However, The *p*-value of the main effect of time tended to be significant only in the C group (*p* = 0.053). As the main effect of treatment was significant, one-way repeated ANOVA was conducted in both groups separately. One-way repeated ANOVA was significant only in the C group (*p* < 0.05). Tukey’s test revealed that the RT at 15:00 were significantly lower than that of 10:00 in the C group (*p* < 0.05).

### 3.2. Plasma Cortisol

**Figure 5:**
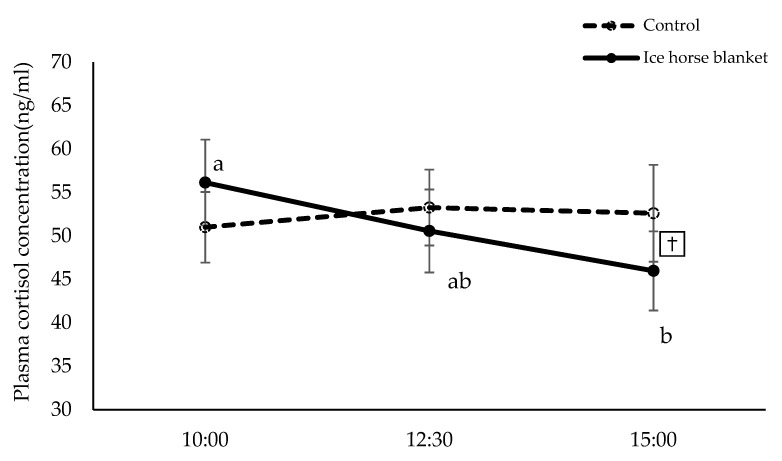
Plasma cortisol concentration. Values are expressed as mean ± SE. Tendency between C and IB at same time point are represented by dagger († *p* < 0.1). Significant differences between each time point within group are represented by different letters (^ab^ *p* < 0.05).

**Figure 6:**
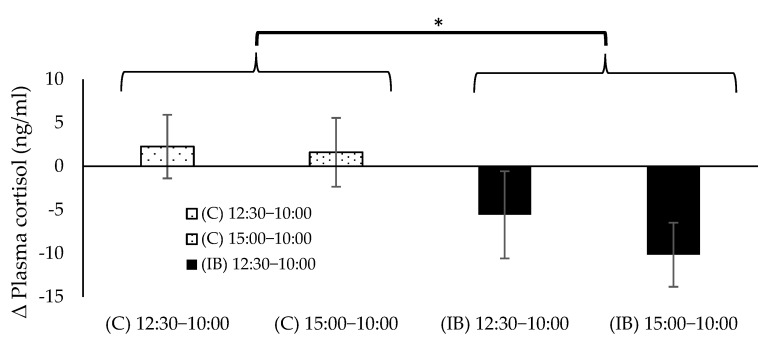
Values are expressed as mean ± SE; Δ plasma cortisol. Significant differences between C and IB at same time point are represented by asterisk (* *p* < 0.05).

Plasma cortisol levels at each time point of C and IB are shown in Figure 5. There was a significant interaction for the cortisol between treatment and time (*p* < 0.05). The simple main effect of treatment was not significant. However, the *p*-value of the main effect of treatment tended to be significant only at 15:00 (*p* = 0.054). In other words, the cortisol in the IB at 15:00 was tended to be lower than that of C (*p* = 0.054). The simple main effect of time was not significant. However, the *p*-value of the main effect of time tended to be significant only in the IB group (*p* = 0.058). In the IB group, the cortisol at 15:00 was significantly lower than that at 10:00 (*p* < 0.05). Δ plasma cortisol at each time point of C and IB groups during the study is shown in Figure 6. There was no significant interaction for Δ plasma cortisol between treatment and time. The main effect of treatment was significant and Δ plasma cortisol was lower in the IB than C (*p* < 0.05). The main effect of time was not significant.

### 3.3. Correlation between Skin Surface Temperatures of the Three Sites of Horse’s Body (Front Back, Rear Back, Loin) and three Measurement Parameters (HR, RR and Plasma Cortisol)

Correlation between skin surface temperatures of the three sites of horse’s body (front back, rear back, loin) and three measurement parameters (HR, RR, and plasma cortisol) are shown in Table 9. The front back and HR of both groups were significantly correlated (R = 0.237, *p* = 0.011). The front back and HR of the C group only were not significantly correlated. The front back and HR of the IB group only was significantly correlated (R = 0.390, *p* = 0.0030). The other correlation results were similar with the correlation result between front back and HR.

## 4. Discussion

The thermoneutral zone is the temperature range within which homeothermic animals do not have to expend energy to maintain core body temperature [9]. According to Morgan [29], the thermoneutral zone for horses ranges between a lower critical temperature of 5 °C and a higher critical temperature of 25 °C. Tamb exceeded 25 °C at 10:00, 12:30, and 15:00 in the present study. As a result, the Tamb in the present study was above the horse’s thermoneutral zone, indicating that the horse was under a heat load. The THI was an effective tool for determining livestock response to climate change [30]. In the present study, THI was found to be between 76 and 78. There are, however, few studies on THI in horses. St-Pierre et al. [31], reported that the THI threshold for dairy cows has been estimated to be around 70. Chaiyabutr et al. [5], who conducted a study on the effects of heat on milk production and fluid regulation in Holstein cattle in a tropical environment, reported that cattle experience mild heat stress between THI 72-74 and are exposed to severe heat stress resulting in decreased milk production at THI 75. Humans are believed to begin feeling stressed at THI 75, according to the Japan Weather Association (JWA) [32]. The present study’s measured conditions were deemed stressful for both humans and dairy cattle. There is no doubt that the climatic conditions in the present study were quite severe for lactating cows and humans, though it is unknown how stressful they were for the horses.

Generally, horses can develop colic if their bodies have been overcooled. The temperature of the cooling site was kept at around 20 °C in the present study to avoid overcooling the site. The horses were kept cooling the three sites of their bodies (the front back, the rear back, and the loin), as the three sites have anatomically blood vessels (capillaries, right azygos vein, caudal vena cava, intercostal arteries, and intercostal veins) and can place objects stably. However, the cooling levels at the three sites were slightly different. As shown in Table 3, the front back’s skin surface temperature was colder than those of the rear back and loin. The blanket displacement was greater at the rear than at the front of the torso, resulting in inadequate cooling of the rear back and loin. While we anticipated that skin surface temperature would also decrease in other sites than the cooled areas, there were no sites with a significant decrease or tendency to decrease outside the three cooled areas. As the ice hose blanket in the present study did not indicate cooling effect outside of the cooled area, it is an inferior method to water in effectiveness.

The skin surface temperatures of fore-cannon and rear-cannon at 12:30 were significantly higher than those at 10:00 in both groups (*p* < 0.05). In horses, the limbs are the area that requires the most extensive care. However, the skin surface temperatures were elevated in both groups. The Limbs cooling with running water is often carried out on wash ruck for conventional daily management of horses and the method could most efficiently decreased skin surface temperature in leisure horses [16]. However, generally, one-time use of the method results in the limb’s temperature rising again over time. Additionally, there are few reports on an effective method for cooling the limbs in stalls, including the present study. Therefore, future studies to develop an effective method for cooling the limbs in stalls are necessary.

As shown in Table 6, the HRs at 12:30 and 15:00 were significantly lower than that of 10:00 in the IB group (*p* < 0.05). Rammerstorfer et al. [33] indicated that the average HR for mature reining horses was 28–34 bpm in thermoneutral conditions. HR increases when the sympathetic nervous system is dominant and decreases to return to the normal value when the parasympathetic nervous system is dominant [34]. In stress situations, the sympathetic nervous system is dominant [35]. HRs were approximately 37 bpm at 10:00, 37 at 12:30, and 36 bpm at 15:00 in the C group, whereas they were approximately 38 bpm at 10:00, 34 bpm at 12:30, and 33 bpm at 15:00 in the IB group. As a result of using the ice blanket, HRs in the IB group were closer to the normal range for horses. RRs in the IB at 12:30 and 15:00 were significantly lower than that of 10:00 (*p* < 0.05). According to Holcomb et al. [9], the average RR for horses in thermoneutral conditions is 10–14 bpm, though the horse breed was not specified. Holcomb et al. [9] showed that the RR of horses was 31.7 bpm in the hot environment at Tamb 37.9°C. At 10:00, 12:30, and 15:00, the C group’s RRs were approximately 32, 31, and 30 bpm. In the IB group, they were approximately 33, 23, and 21 bpm. As a result of an effective cooling process, RRs in the IB group approached the normal value.

RT generally represents the deep body temperature of the animal. Contrary to initial expectations, RTs in the C were significantly lower than those of IB group over the entire measurement period in the present study. RT of the C group were lower at 15:00 than that at 10:00. Although these significant differences occurred, the difference between C and IB was less than 0.1 °C. In the C group, the difference between 10:00 and 15:00 was approximately 0.12 °C. The maximum permissible error for the thermometer used in the present study, according to the manufacturer’s manual, is ±0.1 °C when the device was measured in a thermostatic bath at a room temperature of 23 °C. Given these considerations, the present study’s differences in RT were within the error range. The fact that there was a statistically significant difference in RT at 10:00, when the two groups were not wearing anything, can be considered a difference unaffected at least by the presence or absence of an ice blanket.

As shown in Figure 5, the cortisol at 15:00 was significantly lower than that at 10:00 in the IB group (*p* < 0.05). Cortisol increases in stressed humans [36]. Similarly, cortisol levels were increased in horses during stressful transport [37,38]. Cortisol levels, on the other hand, decrease following a stress-free treatment, such as moving horses from stalls to paddocks [39]. Plasma cortisol levels in the C group were approximately 52.1, 54.6, and 53 ng/mL at 10:00, 12:30, and 15:00 in the present study. Plasma cortisol levels in the IB group, on the other hand, were 55.6, 50.1, and 48.2 ng/mL. In horses, the plasma cortisol level exhibits a circadian rhythm that is higher in the morning and lower at night [40]. Between 12:30 and 15:00, its concentration gradually decreases. Thus, the present study’s findings indicated that the use of the ice blanket maintained normal diurnal variation in cortisol levels.

Takahashi et al. [8], who compared five cooling methods in Thoroughbred horses after strenuous exercise in hot and humid environments, indicated that the cooling methods using the water or fans reduced the RT after 30 min. It was reported by Holcomb et al. [9] that the skin surface temperature, RR and RT of horses (four Quarter horses and eight Thoroughbred horses) were lower in pens with shade than in pens without shade. However, Holcomb et al. [6], found no difference in skin surface temperature, RR, RT, and serum cortisol between horses with and without shade in the study using 18 Thoroughbreds, 13 Quarter horses, an Arabian horse, a Warmblood, and 3 Standardbreds. Under hot conditions, mist curtains or shade in the paddock had no effect on the HR of Warmblood horses [7]. The above study on horses showed that water and fans were effective in reducing heat stress, but shade was effective or ineffective, and mist curtains were ineffective. Numerous research reports on bovines have been published. According to Chaiyabutr et al. [5], evaporative cooling has no effect on the RR, RT, or plasma cortisol levels of crossbred Holstein cattle in a tropical environment. Sprinklers do not reduce RR in Holstein cattle kept in the hot environment [12]. As described in Chanchai et al. [11], cooling with a mist fan decreased RR and RT in cattle containing 87.5% Holstein genes in a tropical environment. These studies show that mist fans, but not evaporative cooling or sprinklers, are effective in cattle.

Wang et al. [41] demonstrated that using a fan device, evaporative cooling clothing, or liquid cooling clothing, the surface temperature of non-exercising humans decreased. The vest equipped with small fans lowered the skin surface and core temperatures of workers [42]. While the ice vest at about 23 °C did not change mean surface temperature or core temperature of humans during exercise, it decreased a part of the skin surface temperature [21]. A 15 °C ice vest reduced perceptual strain but had no effect on HR or surface temperature in humans during exercise [20]. Hasegawa et al. [22] reported that the ice vest expected to be about 16 °C or lower significantly reduced mean skin temperature, HR, and RT in humans in warm and humid conditions. These reports of cooling vests being effective in humans served as a major source of inspiration for the present study’s methodology. As a result, the ice horse blanket was effective in cooling horses staying in their stalls. The present study’s methodology was superior to others, most likely because it allowed for gentle and prolonged cooling only of the horses’ backs to loin without the use of water or fans. Water could not be used in stalls and fans are inadequate for cooling horses. Cooling the belly of a horse is generally not good. Basically, conventional methods would cool the horse’s entire body. In addition, the methods would be rapid and brief cooling. There is no doubt that the ice horse blankets in the present study reduced heat stress in the horses in the stall. Applying this methodology should enable effective reduction of heat stress not only in horses but also in other mammals kept in barns.

The present study has a limitation in that the limbs could not be effectively cooled. Additionally, it is necessary to investigate whether the ice blanket can be used to effectively cool horses immediately after exercise. Moreover, additional research is needed to compare the cost of cooling ice packs with other methods.

## 5. Conclusions

The ice horse blanket used in the present study decreased the HR, RR, skin surface temperature, and plasma cortisol level. This method effectively alleviated heat stress in the stalls, where horses spend the majority of their day. The blanket used in the present study had the advantage of allowing for gentle cooling of the horse’s body without the use of water or fans. Applying this methodology should enable effective reduction of heat stress not only in horses but also in other mammals kept in barns.

## Figures and Tables

**Table 1:** Mean ± SE of ambient temperature (Tamb), relative humidity (RH), and temperature–humidity index (THI).

Weather Condition	Mean ± SE, (*n* = 19)					
	(C)10:00	(C)12:30	(C)15:00	(IB)10:00	(IB)12:30	(IB)15:00
Tamb (°C)	26.5 ± 0.4 ^a^	27.9 ± 0.5 ^b^	27.4 ± 0.5 ^ab^	26.2 ± 0.5 ^a^	27.6 ± 0.6 ^b^	27.1 ± 0.6 ^ab^
RH (%)	71.0 ± 1.8 ^a^	67.2 ± 1.7 ^b^	69.2 ± 1.6 ^ab^	70.9 ± 2.0 ^a^	67.6 ± 1.9 ^b^	69.4 ± 1.9 ^ab^
THI	75.7 ± 0.6 ^a^	77.8 ± 0.7 ^b^	77.3 ± 0.7 ^b^	75.5 ± 0.6 ^a^	77.3 ± 0.8 ^b^	76.9 ± 0.9 ^b^

Significant differences between each time point within group are represented by different letters (^ab^ *p* < 0.05).

**Table 2:** Statistics of ambient temperature (Tamb), relative humidity (RH), and temperature–humidity index (THI).

Weather Condition	Statistics		
	Treatment × Time	ME in Time	ME in Treatment
Tamb (°C)	NS	*p* < 0.05	NS
RH (%)	NS	*p* < 0.05	NS
THI	NS	*p* < 0.05	NS

ME; main effect, NS; not significant.

**Table 3:** Mean ± SE of skin surface temperature.

Skin Surface Temperature (°C)	Mean ± SE, (*n* = 19)					
	(C)10:00	(C)12:30	(C)15:00	(IB)10:00	(IB)12:30	(IB)15:00
Head	31.6 ± 0.5	32.0 ± 0.8	31.7 ± 0.7	31.7 ± 0.8	32.0 ± 0.7	32.0 ± 0.7
Neck	32.0 ± 0.5	32.7 ± 0.7	31.6 ± 0.6	31.6 ± 0.8	32.1 ± 0.6	31.8 ± 0.7
Withers	33.3 ± 0.6	33.4 ± 0.8	33.0 ± 0.7	33.0 ± 0.6	32.4 ± 0.6	32.3 ± 0.6
Shoulder	33.0 ± 0.5	33.1 ± 0.6	32.8 ± 0.6	32.7 ± 0.6	32.3 ± 0.5	32.2 ± 0.5
Front back	32.9 ± 0.6	33.8 ± 0.8	33.2 ± 0.8	32.7 ± 0.6^a^	31.5 ± 0.5 *^ab^	30.9 ± 0.6 *^b^
Front abdomen	32.9 ± 0.5	33.0 ± 0.6	32.5 ± 0.6	32.8 ± 0.6	32.2 ± 0.7	32.3 ± 0.6
Rear back	33.0 ± 0.6	33.7 ± 0.9	33.2 ± 0.8	32.5 ± 0.6	31.5 ± 0.5 *	31.1 ± 0.5 *
Rear abdomen	32.5 ± 0.6	33.2 ± 0.6	32.8 ± 0.7	32.8 ± 0.6	32.6 ± 0.4	32.3 ± 0.5
Loin	32.5 ± 0.5	33.7 ± 0.8	33.3 ± 0.9	32.3 ± 0.6	31.6 ± 0.6 *	31.2 ± 0.5 *
Thigh	32.3 ± 0.5	32.7 ± 0.7	32.3 ± 0.7	32.2 ± 0.6	32.2 ± 0.4	32.1 ± 0.5
Buttock	32.2 ± 0.5	32.9 ± 0.8	32.4 ± 0.8	32.3 ± 0.7	32.2 ± 0.5	32.1 ± 0.5
Fore-cannon	31.8 ± 0.8 ^a^	34.7 ± 1.1 ^b^	33.5 ± 1.2 ^ab^	31.8 ± 1.0 ^a^	33.9 ± 0.9 ^b^	33.0 ± 1.0 ^ab^
Rear-cannon	32.3 ± 0.9 ^a^	35.0 ± 1.1 ^b^	33.9 ± 1.2 ^ab^	31.9 ± 1.1 ^a^	34.4 ± 0.8 ^b^	33.2 ± 0.9 ^ab^

Significant differences between C and IB at same time point are represented by asterisk (* *p* < 0.05). Significant differences between each time point within group are represented by different letters (^ab^ *p* < 0.05).

**Table 4:** Statistics 1 of skin surface temperature.

Skin Surface Temperature (°C)	Statistics		
	Treatment × Time	ME in Time	ME in Treatment
Head	NS	NS	NS
Neck	NS	NS	NS
Withers	NS	NS	NS
Shoulder blade	NS	NS	NS
Front back	*p* < 0.05	-	-
Front abdomen	NS	NS	NS
Rear back	*p* < 0.1	-	-
Rear abdomen	NS	NS	NS
Loin	*p* < 0.05	-	-
Thigh	NS	NS	NS
Buttock	NS	NS	NS
Fore-cannon	NS	*p* < 0.05	NS
Rear-cannon	NS	*p* < 0.05	NS

ME; main effect, NS; not significant.

**Table 5:** Statistics 2 of skin surface temperature.

Skin surface Temperature (°C)	Statistics				
	(10:00) SME in Treatment	(12:30) SME in Treatment	(15:00) SME in Treatment	(C) SME in Time	(IB) SME in Time
Head	-	-	-	-	-
Neck	-	-	-	-	-
Withers	-	-	-	-	-
Shoulder blade	-	-	-	-	-
Front back	NS	*p* < 0.05	*p* < 0.05	NS	*p* < 0.05
Front abdomen	-	-	-	-	-
Rear back	NS	*p* < 0.05	*p* < 0.05	NS	*p* < 0.1
Rear abdomen	-	-	-	-	-
Loin	NS	*p* < 0.05	*p* < 0.05	NS	NS
Thigh	-	-	-	-	-
Buttock	-	-	-	-	-
Fore-cannon	-	-	-	-	-
Rear-cannon	-	-	-	-	-

SME; simple main effect, NS; not significant.

**Table 6:** Mean ± SE of heart rate (HR), respiratory rate (RR), and rectal temperature (RT).

Physiological Measures	Mean ± SE, (*n* = 19)					
	(C)10:00	(C)12:30	(C)15:00	(IB)10:00	(IB)12:30	(IB)15:00
HR (bpm)	36.6 ± 0.9	36.6 ± 1.2	36.0 ± 1.0	38.1 ± 1.1 ^a^	33.9 ± 0.5 *^b^	32.9 ± 0.7 *^b^
RR (bpm)	32.4 ± 1.7	30.6 ± 1.5	30.0 ± 1.1	33.3 ± 2.5 ^a^	23.1 ± 1.4 *^b^	21.6 ± 1.5 *^b^
RT (°C)	37.32 ± 0.05 ^a^	37.23 ± 0.04 ^ab^	37.20 ± 0.04 ^b^	37.36 ± 0.05 *	37.38 ± 0.04 *	37.32 ± 0.04 *

Significant differences between C and IB at same time point are represented by asterisk (* *p* < 0.05). Significant differences between each time point within group are represented by different letters (^ab^ *p* < 0.05).

**Table 7:** Statistics 1 of heart rate (HR), respiratory rate (RR), and rectal temperature (RT).

Physiological Measures	Statistics		
	Treatment × Time	ME in Time	ME in Treatment
HR (bpm)	*p* < 0.05	-	-
RR (bpm)	*p* < 0.05	-	-
RT (°C)	NS	*p* < 0.1	*p* < 0.05

ME; main effect, NS; not significant.

**Table 8:** Statistics 2 of heart rate (HR), respiratory rate (RR), and rectal temperature (RT).

Physiological Measures	Statistics				
	(10:00) SME in Treatment	(12:30) SME in Treatment	(15:00) SME in Treatment	(C) SME in Time	(IB) SME in Time
HR (bpm)	NS	*p* < 0.05	*p* < 0.05	NS	*p* < 0.05
RR (bpm)	NS	*p* < 0.05	*p* < 0.05	NS	*p* < 0.05
RT (°C)	-	-	-	-	-

SME; simple main effect, NS; not significant.

**Table 9:** Correlation between skin surface temperatures of the three sites of horse’s body (front back, rear back, loin) and 3 measurement parameters (HR, RR, and plasma cortisol).

Measures	Front Back and HR			Rear Back and HR			Loin and HR		
	Both	C	IB	Both	C	IB	Both	C	IB
Correlation	0.237	0.0550	0.390	0.232	0.0470	0.396	0.235	0.0830	0.365
*p*-value	0.011	NS	0.0030	0.013	NS	0.0020	0.012	NS	0.0050
Measures	Front back and RR			Rear back and RR			Loin and RR		
	Both	C	IB	Both	C	IB	Both	C	IB
Correlation	0.317	0.135	0.386	0.295	0.344	0.344	0.257	0.177	0.298
*p*-value	0.0010	NS	0.0030	0.0010	NS	0.0090	0.0060	NS	0.025
Measures	Front back and cortisol			Rear back and cortisol			Loin and cortisol		
	Both	C	IB	Both	C	IB	Both	C	IB
Correlation	0.271	0.211	0.355	0.213	0.157	0.288	0.213	0.160	0.281
*p*-value	0.0040	NS	0.0070	0.023	NS	0.030	0.023	NS	0.034

NS: not significant.

## Data Availability

The data presented in this study are available on request from the corresponding author.
